# A Clearer View of TCE: Evidence Supports Autoimmune Link

**DOI:** 10.1289/ehp.117-a210a

**Published:** 2009-05

**Authors:** Bob Weinhold

More than 80 known or suspected autoimmune disorders—such as Crohn disease, multiple sclerosis, and rheumatoid arthritis—affect 5–8% of the U.S. population, according to the National Institute of Allergy and Infectious Diseases. The underlying causes of these disorders remain largely unknown, but one agent suspected to play a role is trichloroethylene (TCE), a solvent widely used in industrial and household applications. Researchers from the U.S. Environmental Protection Agency’s National Center for Environmental Assessment and the Medical University of South Carolina searched the scientific literature for studies linking TCE with selected immunologic connections, including immunosuppression, hypersensitivity, and autoimmune-related effects ***[EHP 117:696–702; Cooper et al.]***. On the basis of their review, the authors concluded that the evidence to date in mice and humans supports an etiologic role of TCE in autoimmune disorders.

Substantial evidence from mechanistic, clinical, and epidemiologic studies indicates that exposure to TCE and/or its metabolites (including chloral hydrate, trichloracetic acid, trichloracetaldehyde hydrate, and dichloracetyl chloride) could influence the incidence of autoimmune disorders. Research on autoimmune mouse models, including the MRL^+/+^ lupus mouse, has provided strong and consistent support for a role of TCE; this has included studies of exposures at environmentally relevant concentrations through multiple routes (inhalational, dermal, and oral). Studies of humans with high occupational or environmental exposures have also shown links between TCE and inflammatory immune responses, systemic sclerosis (scleroderma), and a severe generalized hypersensitivity skin disorder.

However, the authors also point out major gaps in our knowledge of TCE’s effects on the immune system. In particular, data pertaining to measures of immunosuppression in humans are very limited, and potential effects of age or sex on susceptibility to autoimmune-related effects of TCE exposures, as well as effects of variation in exposure dose, timing, and duration, have yet to be established.

Because individual autoimmune diseases are relatively rare, it is difficult to assemble enough cases to conduct adequately powered epidemiologic research. However, the authors assert that the findings of recent experimental and observational studies of TCE provide a strong rationale for developing multisite collaborations to address the potential influence of TCE and other solvents on the incidence of autoimmune disorders. Such research would be facilitated by the establishment of state and national autoimmune disease registries.

